# A Firing Cannon: Bilateral Lower Limb Ischemia as a Manifestation of Cardiac Myxoma

**DOI:** 10.1055/a-2489-6334

**Published:** 2024-12-24

**Authors:** Mohamed Elbayomi, Raphael Groß, Pathare Presheet, Abbas Agaimy, Mattias May, Micheal Wayend, Christian Heim

**Affiliations:** 1 Department of Cardiac Surgery, Friedrich-Alexander-University Erlangen-Nuremberg, Erlangen, Germany; 2 Institute of Pathology, Friedrich-Alexander-University Erlangen-Nuremberg, Erlangen, Germany; 3 Department of Interventional Radiology, Friedrich-Alexander-University Erlangen-Nuremberg, Erlangen, Germany; 4 Cardiac and Vascular Surgery, Klinikum Bayreuth, Medical Campus Oberfranken of Friedrich Alexander University, Germany

**Keywords:** left atrium myxoma, surgery, cardiopulmonary bypass, case report, interventional radiology

## Abstract

**Background** Cardiac myxomas are the most common primary cardiac neoplasms.

**Case Description** We present a case of a middle-aged lady with cardiac myxoma in her left atrium awaiting semi-elective surgery. During the preoperative period, the patient presented emergently with acute bilateral lower limb ischemia. This lady was fortunate to have these emboli fired towards a tissue with ischemia tolerance capacity. If these emboli had been directed toward the central nervous system, this patient would have experienced devastating complications and even death.

**Conclusion** This presentation opens the debate on the timing of surgical intervention in myxoma patients.

## Introduction

A primary cardiac tumor is a rare disease, with an incidence of 1.38 per 100,000 persons per year. 1 Approximately 90% of primary cardiac tumors are benign in nature. 2 The most prevalent of them is left atrial myxoma. Myxomas are usually described as a “ball valve” obstruction in the left atrium and are often associated with multiple syncopal episodes. 3 Systemic embolization occurs in about 30% of the cases of atrial myxoma. In the local examination of the heart, an early diastolic murmur could usually be heard on the fifth intercostal space. The symptoms typically worsen in the upright position and improve when lying down. Interleukin-6 (IL-6) produced by the tumor cells may predispose to constitutional symptoms such as fever and weight loss. Approximately 7% of all cardiac myxomas are associated with the Carney complex, and chromosomal abnormalities involving chromosomes 2p, 12, and 7q have been reported in this regard. However, no genetic abnormalities at these loci could be detected in sporadic cases. 4

This article reports a left atrial myxoma in a young female who presented with bilateral lower limb ischemia.

## Case Presentation

**Video 1** A transthoracic 4-chamber view reveals the left atrial mass protruding through the mitral valve into the left ventricle during diastole.


**Video 2** The left atrial mass attaches to the atrial septum with heterogeneous echogenicity in a transthoracic 4-chamber view.


We present a 33-year-old Caucasian smoker female (153 cm, 58 kg). Her medical history included endometriosis. A few days prior, she had been diagnosed with left atrial myxoma. The patient was scheduled within 1 week. During this waiting period, she presented to our institution's emergency department (ED) complaining of worsening bilateral lower limb pain. Upon admission, the patient reported dyspnea with ordinary activity (NYHA III) for several months. She denied chest pain, fever, abdominal pain, nausea, or weight loss.

During the physical examination, the patient presented normotensive (114/65 mmHg), with a rhythmic heartbeat (76 BPM), and normophonetic heart sound, without any existing heart murmurs. There were no signs of heart failure and no focal neurological deficit. Pupils were isocoric and reactive to light and accommodation. There was no neck vein distention, and carotid artery auscultation did not reveal any bruits. The abdomen was soft and non-tender, non-distended, with normal bowel sounds in four quadrants. The femoral and radial arterial pulses were bilaterally palpable. However, the arteriae tibialis posterior and dorsalis pedis were not palpable bilaterally. The ankle-brachial pressure index (ABPI) was about 0.9 on the right side and 0.8 on the left side of the body. There was no lower limb edema noticed. ECG showed a biphasic P atrial wave pattern “P mitrale” consisting of a notch (double hump) near its peak, as seen in Lead II. Transthoracic echocardiography (TEE) demonstrated a normal left ventricular ejection fraction with no wall motion abnormalities, and a large left atrial mass (5.1 × 2.5 × 2.8 cm) that protruded through the mitral valve into the left ventricle, which predisposed moderate mitral valve stenosis with a mean pressure gradient of 10 mmHg ( Videos 1 and 2 ).

**Fig. 1:**
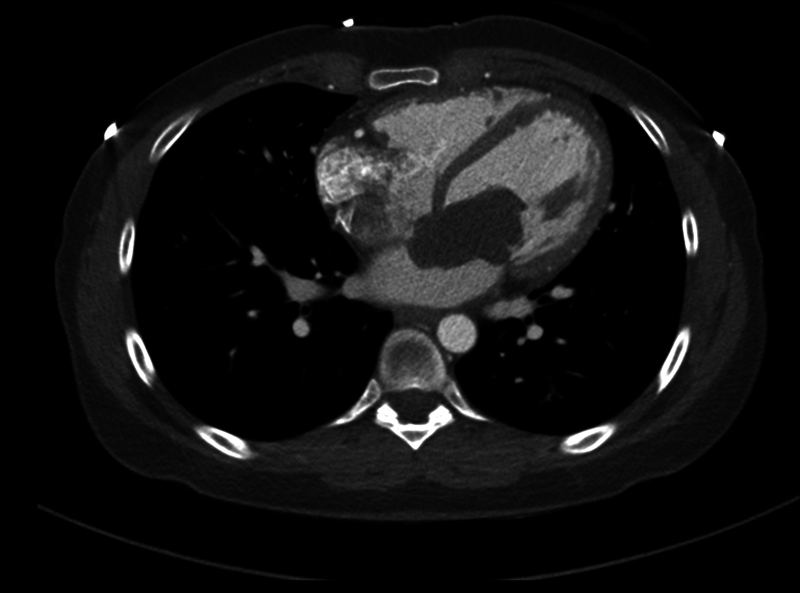
A computed tomography axial view demonstrating the huge left atrial mass protruding to the left ventricle through the mitral valve.

A chest computed tomography (CT) scan demonstrated the intracardiac pedunculated mass in the left atrium, with the tumor protruding into the left ventricle through the mitral valve ( Fig. 1 ).

Lower limb Doppler sonography yielded an occlusive thrombus of the popliteal artery in both the lower limbs. Still, both the limbs were still warm and perfused through collaterals; the patient fulfilled the criteria of Rutherford classification class I.

**Fig. 2:**
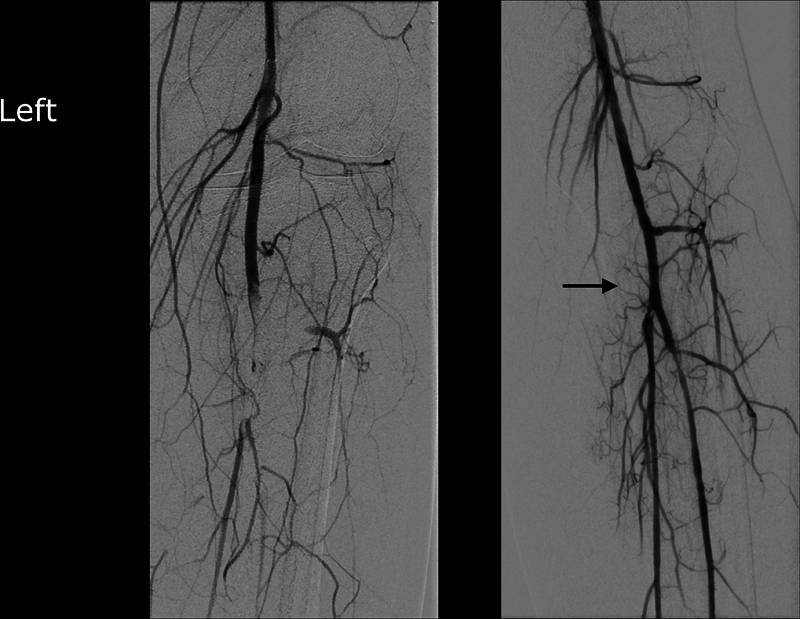
Catheter angiography in the digital subtraction technique showed embolic material in the P3 segment of the popliteal artery, in the transverse section of the anterior tibial anterior, and in the tibiofibular tractus (TFT) in the left lower limb. White chewy material was removed by aspiration thrombectomy until the lower limb perfusion was compensated. Postinterventional angiography (arrow) yielded complete recanalization of the occlusion.

**Fig. 3:**
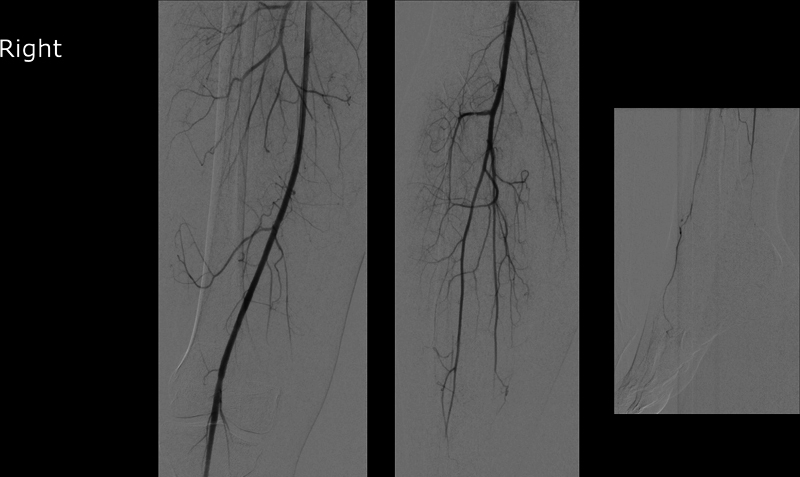
Small peripheral emboli were also found at the level of the ankle on the right side in an additional angiogram of the right side. An interventional approach was waived due to the peripheral location and the preserved perfusion of the plantar arch.

The patient underwent an urgent catheter angiography by digital substruction technique, which yielded multiple emboli in the P3 segment of the popliteal artery, in the transverse section of the anterior tibial artery, and in the tibiofibular tractus. White gelatinous material was removed by aspiration thrombectomy from the culprit lesions of the left lower limb. Surprisingly enough, the microscopic specimen of the aspirated material showed cellular structure and matrix of a myxoma. The left lower limb perfusion was restored. In an additional angiogram of the right lower limb, small peripheral emboli were found at the ankle level; an interventional approach was waived due to the peripheral location of the lesion and the preserved perfusion of the planter arch ( Figs. 2 and 3 ).

**Fig. 4:**
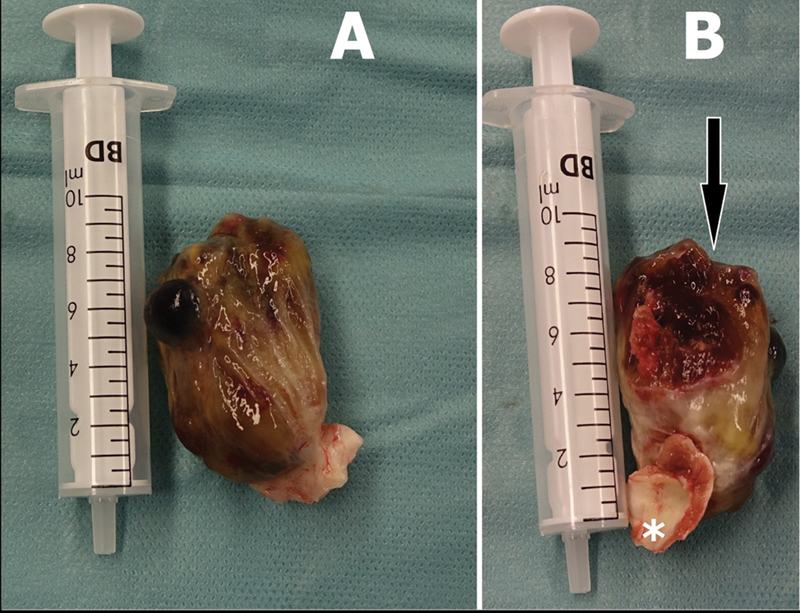
Intraoperative pictures of the myxoma after excision (syringe for scale). ( **A** ) A large body of the myxoma with a disproportionately small pedicle at the area of attachment to the left atrium/atrial septum. ( **B** ) Arrow indicates the area of the myxoma that was embolized. Asterisk shows the base of the myxoma with part of the left atrium/atrial septum.

Cardiac CT showed no obstructive coronary artery disease. A preoperative workup was done. The patient underwent surgical excision of the left atrial mass with median sternotomy, as a minimally invasive approach would need peripheral cannulation for extra-corporal circulation. After hemi-sternotomy, she was heparinized with checking of activated clotting time. Aortic-bicaval cannulation was installed, and cardiopulmonary bypass (CPB) was established. After an aortic cross-clamp was applied, a satisfactory diastolic arrest could be achieved using an antegrade infusion of normothermic blood cardioplegic solution in the aortic root, after which a right atriotomy was performed. An incision at the fossa ovalis was performed to access the left atrium revealing a large mobile mass; it was found to be attached to the left atrial septum via a stalk at the level of the fossa ovalis. Due to the significant size of the mass, a wider incision of the fossa ovalis was made to excise the myxoma along with its stalk and ensure a precise margin of excision ( Fig. 4 ). The tumor was then carefully resected with a high level of care to prevent its fragmentation. After careful inspection of the atria to exclude any residual mass, a bovine pericardial patch was used to reconstruct the septum, and the right atriotomy was closed. The root was vented. The heart was de-aired, and the aortic cross-clamp was removed. The weaning of the cardiopulmonary bypass was routinely performed after the spontaneous return of the sinus heartbeats. Protaminized decannulation was achieved, and hemostasis was secured. The chest was then closed in layers in the usual manner. The patient tolerated the following procedures well and was shifted to the intensive care unit. She was extubated after 4 hours and discharged to normal for postoperative care after 12 hours.

Postoperative EKG showed a normal sinus rhythm. Postoperative transesophageal echocardiography showed an left ventricle ejection fraction (LVEF) of 50% with no residual mass in the left atrium, normal mitral valve, no patent foramen ovale, and no pericardial effusion. The patient tolerated the procedure well and had an uneventful postoperative course.

The histopathological examination of the mass showed stellated fusiform and polygonal cells immersed in an amorphous myxoid matrix. CD31 and CD34 as immunohistochemical markers for myxoma were present.

Calretinin staining of the mass was strongly positive, confirming the presence of cardiac myxoma cells. Cultures from the mass tissue yielded no growth of any bacterial organism.

The patient was discharged uneventfully and is being monitored in the outpatient clinic. From the patient's perspective, on follow-up, the patient was satisfied with her decision to undergo surgery to excise the cardiac myxoma. Her physical activity levels increased post-interventionally, and she returned to everyday life.

## Discussion

The current knowledge suggests that cardiac myxomas might originate from embryonic entrapped mesenchymal multipotent progenitor cells. There is evidence that the cytokine vascular endothelial growth factor is involved in tumor growth and angiogenesis. Histological myxomas consist of a myxoid matrix composed of an acid mucopolysaccharide-rich stroma. On the macroscopic level, typical myxomas are pedunculated and gelatinous in consistency. The myxoma surface was studied in a large series of cases; in one-third of the patients, it was friable or villous, and smooth in the other two-thirds of the cases. The villous form myxomas had a surface that consisted of multiple, fine, villous gelatinous and fragile extensions with a tendency to fragment spontaneously. The villous-type surface myxomas tend to cause embolization. 5

In terms of myxomas, despite the benign nature of the tumor, surgical excision is highly recommended to prevent complications such as sudden cardiac death or arterial embolization. Although the timing of the surgery is not clearly defined, the patients can experience a major compilation while waiting. Hence some researchers suggest surgery should be performed urgently once it has been identified that the patient has a myxoma large enough to predispose to compilations. 6

Echocardiography is a readily accessible, noninvasive technique for initial evaluation. Usually, TEE is sufficient to confirm the diagnosis. However, transesophageal echocardiography may be more informative. The superiority of this diagnostic method is due to the proximity of the esophagus to the left atrial chamber and the lack of intervening lungs and bones. Cardiac magnetic resonance imaging (MRI) and CT can also provide noninvasive, high-resolution heart images. 7 MRI is generally preferred. It can furnish detailed anatomic images that reflect the chemical environment within a neoplasm, hence offering clues as to the type of tumor present. Still, the accessibility of MRI is limited in many territories around the world. CT can yield a diagnosis in case MRI is unavailable or contraindicated. 8 9

A transvenous biopsy is an invasive diagnostic utility rarely used due to the possibility of embolization. However, a biopsy is considered reasonable for other cardiac tumors if potential benefits are deemed sufficient to outweigh potential risks.

Surgical removal of the tumor is the treatment of choice; no effective medical treatment stops the tumor growth. Surgery should be performed as soon as possible. If surgery is delayed, the patient may suffer devastating, irreversible central nervous system damage due to the potential of left atrial myxoma to predispose an embolization, especially in an era where cardiac surgery is advanced and can be performed with significantly low morbidity and mortality rates. We conducted an embolectomy before the cardiac procedure on our patient due to evidence of lower limb ischemia, which prompted us to prioritize the culprit lesion in the lower limb. We exercised utmost caution while manipulating the heart during surgery, meticulously dissecting the tumor to prevent a subsequent embolization wave that could result in severe postoperative morbidity. That raises a question: Should the surgical rule “hit hard and early” be applied to managing myxoma patients to prevent such a complication?

Surgical treatment is curative, and incidence of recurrence is very low in sporadic isolated myxomas. However, recurrence can be higher in diseases with genetic etiology, such as the Carney complex. The PRKAR1A gene has been identified in patients with Carney complex. For this reason, it might be beneficial for the patient to undergo genetic counseling for the PRKAR1A gene in order to identify the follow-up plan. 10

In one large series, 5% of the participants developed recurrent myxoma, suggesting the need for careful follow-up. Development of a second primary myxoma may be more common in patients with a family history of myxoma. 5 Although surgical resection is curative and the recurrence rate is low, patients may benefit from serial echocardiograms in outpatient settings. 11

## Conclusion

If surgery is indicated, any delay should be avoided. Prompt resection can prevent devastating and irreversible complications such as stroke.
